# Prevalence of Nocturnal Enuresis among Saudi Children Population

**DOI:** 10.7759/cureus.6662

**Published:** 2020-01-15

**Authors:** Elham H Alhifthy, Lura Habib, Azahir Abu Al-Makarem, Maryam AlGhamdi, Doaa Alsultan, Fatemah Aldhamer, Rawan Buhlagah, Fatimah M Almubarak, Eatesam Almufadhi, Ghaida M Bukhamsin, Maria H Zadah

**Affiliations:** 1 Clinical Sciences, Pediatrics, Developmental and Behavioural Pediatrics, Princess Nourah Bint Abdulrahman University, College of Medicine, Riyadh, SAU; 2 Medicine, Taif University, Taif, SAU; 3 Medicine, Umm Alqura University (UQU), Makkah, SAU; 4 Medicine, Aljouf University, Al-Jouf, SAU; 5 Medicine, King Faisal University, Riyadh, SAU; 6 Medicine, Princess Nourah University, Riyadh, SAU; 7 Medicine, Al Jouf University, Al-Jouf, SAU; 8 Medicine, Princess Nourah Bint Abdulrahman University, Riyadh, SAU

**Keywords:** risk factors, provided treatment, nocturnal enuresis

## Abstract

Background

Nocturnal enuresis (NE) is the involuntary urination that occurs while asleep after an age when bladder control at night is expected. It has a global incidence of 1.4%-28% among 6-12 years old children. The aim of this study is to show the prevalence, risk factors, types of provided treatment of enuresis among studied children in Kingdom of Saudi Arabia (KSA).

Methods

A cross-sectional descriptive study was carried out among Saudi children, 3-12 years of age, from different cities in Saudi Arabia, during the period from 20 October to 20 November 2019. Data was collected by using a pre-designed questionnaire that was distributed online and included questions designed to fulfill the study objectives.

Results

This study reported that 31.2% of Saudi children of the chosen ages are suffered from enuresis, the majority occurred at day and night by 55.1% while 43.9% occurred only at night. Participants described types of provided treatment as follows: behavioral modification was the most commonly used by 31.6% followed by pharmacological intervention (29.6%), bed-wetting alarm (6.8%), exercises to strengthen the bladder muscles (6.2%) and surgical intervention reported by 1.5% only. It was found that the improvement of enuresis on treatment occurred in 43.6% of cases. There was a significant reduction of the prevalence of NE with age (peak is 63.6% in 5-7 years old) but no significant correlation was found with gender (p = 0.104). However, there was a significant correlation with parent having history of NE (p = 0.001).

Conclusion

The study reported that 31.2% of children found to have nocturnal enuresis; 43.9% of those had nocturnal enuresis alone. There were no significant correlations between nocturnal enuresis and child gender while it significantly correlated with child’s age and having a family history of NE. Behavioral modification therapy was the most commonly provided treatment followed by pharmacological intervention; improvement occurred in less than half of the cases with treatment.

## Introduction

Nocturnal enuresis (NE) is the involuntary urination that occurs while asleep after an age when bladder control at night is expected (Involuntary urination that happens during the day is known as diurnal enuresis.) [1]. Nocturnal enuresis is a common problem in children and teenagers. NE is the second most common disorder affecting children 6-14 years of age after allergic disorders [2]. The global incidence of enuresis in children 6-12 years of age was shown to be 15%-25% in one study [3]. While a study in Egypt showed an incidence of 18% among children 9 ± 2 years old [4].

Enuresis may be classified into primary and secondary forms. Primary enuresis is when a child >5 years of age has never achieved a period of complete dryness for six or more months in a row [5]. While secondary enuresis is a condition that develops at least six months or several years after a child has achieved a period of complete dryness.

There are a variety of proposed causes of NE including: low bladder capacity, insufficient antidiuretic hormone production at night, familial/genetic causes, upper airway obstructions or less often structural problems in the urinary tract or nervous system [6]. Other causes include a variety of development, diabetes insipidus, urinary tract infections, stressful circumstances in school or family [7].

Nocturnal enuresis may lead to low self-esteem, a sense of failure, chronic stress and it may affect the child’s social life. It can trigger a range of behavioral, psychological and social problems. Therefore, it is important to identify children at risk and perform therapeutic measures [8].

Nocturnal enuresis is known to have multiple comorbidities which raises the importance of recognizing it and looking for associated symptomatology. Those comorbidities include neuropsychiatric problems like intellectual disability, attention deficit hyperactivity disorder (ADHD), psychological disorders and low self-esteem. Urinary tract infections, obstructive sleep apnea, diabetes, and low hormones as ADH are other disorders known to be associated with NE [3,9].

This study aims to find the prevalence of nocturnal enuresis within children in Saudi Arabia, its risk factors and provided management modalities of enuresis among 3-12-year-old children in different cities in KSA.

## Materials and methods

Participants and methods

A cross-sectional descriptive study was carried out among 2148 Saudi children aged 3-12 years from different Saudi provinces, which represent different localities and cultures of the kingdom. It was conducted during the period from 20 October to 20 November 2019. The sample size of 2148 was calculated according to the sample size using the equation of n = z^2^p (1-p)/e^2 ^(n = sample size, z = level of confidence according to the standard normal distribution, p = estimated proportion of the population that presents the characteristic, and e = tolerated margin of error). A convenient sampling technique was followed.

Data collection

Data was collected using a predesigned online questionnaire distributed to parents/caregivers and included questions designed to fulfill the study objectives.

The questionnaire included questions about the following main items:

· Socio-demographic data of the participants (age, gender, and birth order of the child),

· The personal knowledge of the respondents about nocturnal enuresis and its causes,

· Enuresis-related characteristics like time, relation to sleeping, frequency per week, an improvement in decreasing fluid intake before sleeping, and others,

· Risk factor like a family history of NE in parents or siblings, chronic diseases, anemia, delayed milestones, and others,

· Management modalities used for children who have NE.

Ethical considerations

The questionnaire has a brief introduction explaining its aim and purpose and informing participants that participation is entirely voluntary. No names were recorded in the surveys, neither date of birth nor address has been collected. All answers were kept confidential and safe.

Statistical analysis

Data were analyzed using Statistical Package for Social Sciences (SPSS) version 20 (IBM Corp., Armonk, NY). Descriptive statistics were used for the prevalence and quantitative variables. Risk factors were determined using the X^2^ test (x = sample size mean). P-value of less than 0.05 was considered statistically significant.

## Results

The questionnaire was distributed online through social media. Two thousand one hundred and forty-eight (2148) responses where received and analyzed. This study identified a prevalence of enuresis of 31.2%.

Most children (59.6%) were 5-7 years of age, 58.2% of the sample were males, and 31.4% of studied children were the first child (Table 1). The majority (61.3%) of respondents knew about nocturnal enuresis, and 34.2% of them believed they know its causes. Nineteen percent identified causes of nocturnal enuresis as weakness in the muscles of the lower urinary tract, 9.1% as problems or damage of the urinary tract or nerves that control the urinary system, 8.0% as psychological problems, and 2.8% as urinary tract infection. About a quarter (25.7%) of participants had at least one other child suffering from nocturnal enuresis.

**Table 1:** Child age, sex, birth order, parents’/caregivers’ knowledge about nocturnal enuresis and prevalence of nocturnal enuresis among the studied children

	Frequency	Percent
Child age (in years)		
3-5	556	25.9
5-7	1280	59.6
7-10	216	10.1
>10	94	4.4
Child sex		
Female	898	41.8
Male	1248	58.2
Birth order of the child		
1	674	31.4
2	416	19.4
3	340	15.8
4-5	384	17.9
>5	332	15.5
Participants know about nocturnal enuresis		
No	832	38.7
Yes	1316	61.3
Participants know about its causes		
No	1414	65.8
Yes	734	34.2
Identified causes of nocturnal enuresis (by participants)		
Weakness in the muscles of the lower urinary tract	408	18.9
Problems or damage of the urinary tract or nerves that control the urinary system	196	9.1
Psychological problems	172	8.0
Urinary tract infections	60	2.8
Hereditary	54	2.5
Anemia	12	.6
Irritability	18	.8
Pregnancy and birth-related causes	8	.4
Having a child suffering from nocturnal enuresis		
No	1478	68.8
Yes	670	31.2

Table 2 shows nocturnal enuresis-related characteristics among the studied sample. More than 40% of the children had enuresis at night only, while 55.1% had it during day and night although 78.8% of children improved on decreasing fluid intake before sleeping within 5-7 weeks. The problem caused embarrassment and social shame for 94.3% of studied children, and 76.4% sought medical advice. Of those 29.6% received pharmacological treatment, 31.6% behavioral modification, 6.8% bedwetting alarm, 6.2% by exercise, and only 1.5% had surgery.

**Table 2:** Nocturnal enuresis-related characteristics among the studied cases

Variable	Frequency	Percentage
Time of enuresis		
At night only	294	43.9
Day and night	376	55.1
Improvement of decreasing fluids intake before sleeping	344	51.3
Frequency per week		
1-2	80	11.9
3-4	102	15.2
5-7	528	78.8
Mother keen to wake the child at night to urinate	610	91.0
The problem causes embarrassment and social shame to the child	632	94.3
Seeking medical advice	512	76.4
Type of provided treatment		
Pharmacological treatment	198	29.6
Surgery	10	1.5
Exercises to strengthen the bladder muscles	42	6.2
Bedwetting alarm	46	6.8
Behavioral modification	212	31.6
Improvement of nocturnal enuresis on different types of treatment	262	43.6

This study has shown significant relationship between enuresis and child’s age (P = 0.05) and gestational age (in months) at birth (P = 0.013), type of delivery, hospital admission after delivery, sibling suffering from the same condition, birth order of the child, parents’ history of NE, diabetes, urinary tract infection, psychological problems and delayed milestones (P < 0.05) (Table 3). While there were no significant correlations between nocturnal enuresis and child gender (P = 0.104).

**Table 3:** Risk factors of nocturnal enuresis among the studied children

Variables	Responses	Nocturnal enuresis		Total (N = 2148)	P value
		Yes (n = 670)	No (n = 1478)		
Child age (in years)	3-5	150	408	558	0.05
		22.4%	27.6%	26.0%	
	5-7	426	854	1280	
		63.6%	57.8%	59.6%	
	7-10	64	152	216	
		9.6%	10.3%	10.1%	
	>10	30	64	94	
		4.5%	4.3%	4.4%	
Sex	Female	300	598	898	0.104
		44.8%	40.5%	41.8%	
	Male	370	880	1250	
		55.2%	59.5%	58.2%	
Gestational age (in months)	9	612	1412	2024	0.013
		91.3%	95.5%	94.2%	
	8	32	24	56	
		4.8%	1.6%	2.6%	
	7	18	20	38	
		2.7%	1.4%	1.8%	
	<7	8	22	30	
		1.2%	1.5%	1.4%	
Type of delivery	Vaginal	520	1290	1810	0.002
		77.6%	87.3%	84.3%	
	Cesarean section	150	188	338	
		22.4%	12.7%	15.7%	
Hospital admission after delivery	No	588	1392	1980	0.003
		87.8%	94.2%	92.2%	
	Yes	82	86	168	
		12.2%	5.8%	7.8%	
Sibling suffering from the same condition	No	498	1324	1822	0.006
		74.3%	89.6%	84.8%	
	Yes	172	154	326	
		25.7%	10.4%	15.2%	
Birth order of the child	1	204	472	676	0.002
		30.4%	31.9%	31.5%	
	2	108	308	416	
		16.1%	20.8%	19.4%	
	3	108	232	340	
		16.1%	15.7%	15.8%	
	4-5	164	220	384	
		24.5%	14.9%	17.9%	
	>5	86	264	332	
		12.8%	16.6%	15.5%	
History of parents with same condition during their childhood	No	550	1354	1904	0.001
		82.1%	91.6%	88.6%	
	Yes	120	124	244	
		17.9%	8.4%	11.4%	
The child has chronic illness	No	618	1386	2004	0.210
		92.2%	93.8%	93.3%	
	Yes	52	92	144	
		7.8%	6.2%	6.7%	
Anemia	No	594	1356	1950	0.067
		88.7%	91.7%	90.8%	
	Yes	76	122	198	
		11.3%	8.3%	9.2%	
Parasitic infestation	No	634	1412	2046	0.306
		94.6%	95.5%	95.3%	
	Yes	36	66	102	
		5.4%	4.5%	4.7%	
Diabetes type I	No	630	1438	2068	0.009
		94.0%	97.3%	96.3%	
	Yes	40	40	80	
		6.0%	2.7%	3.7%	
Urinary tract infection	No	610	1444	1054	0.000001
		91.0%	97.7%	95.6%	
	Yes	60	34	94	
		9.0%	2.3%	4.4%	
Psychological problems	No	546	1368	1914	0.000001
		81.5%	92.6%	89.1%	
	Yes	124	110	234	
		18.5%	7.4%	10.9%	
Delayed milestones	No	628	1438	2066	0.005
		93.7%	97.3%	96.2%	
	Yes	42	40	82	
		6.3%	2.7%	3.8%	

## Discussion

Nocturnal enuresis (NE) is the involuntary urination that occurs while asleep after an age when bladder control at night is expected. It is more common in children living in unfavorable social conditions who are under psychosocial stress and is known to affect a child’s psychological state [1,10]. It is commonly identified amongst school-aged children with a significant stressor along with psychosocial problems both for parents and children [1]. Multiple mechanisms have been proposed for NE, including dysfunctional bladder, small functional bladder capacity, abnormal antidiuretic hormone levels, and irregular sleep patterns [11].

This is a cross-sectional study that was conducted among 2148 children in KSA and aimed to show the prevalence, risk factors, provided modalities of treatment for nocturnal enuresis among studied children in KSA.

The prevalence of nocturnal enuresis in children is between 15 and 25% at five years of age, and it goes down as children grow [3]. In this study, the majority of participants had children aged 5-7 years, of whom 63% had NE, while the prevalence significantly decreased to 9.6% in children 7-10 years and 4.5% in children older than 10 years of age (P = 0.05). The prevalence was close to what is reported from Sherah et al. study conducted in Jazan city in Saudi Arabia for children aged 5-12 years which was 76.4% but was higher than those reported from other studies where the overall prevalence of enuresis was found to be 12.95% in children aged 5-16 years from France and 15% in children aged 6-11 years from Saudi Arabia and two studies in Turkey estimated the prevalence to be 12.4 % and 13% [1,12-15]. However, in India, another survey carried out among 1473 children aged between 6 and 10 years reported that the overall prevalence of enuresis was 7.61% [16]. In Iran, a systemic review conducted to estimate the prevalence of enuresis and its related factors among Iranian children found that the prevalence of enuresis among all children was estimated as of 11.01% [17].

Most reported enuresis cases in our study had it at day and night 55.1%, and 43.9% occurred at night only. However, Sherah et al. and Sarici et al. reported that daytime enuresis was seen in only 14.29% and 18% of cases, respectively, of children of school-age [12,18].

The causation of enuresis is generally multifactorial and is the result of the interaction of physical and psychological factors. Participants believed that enuresis could be caused by weakness in the muscles of the lower urinary tract by 18.9%, problems or damage of the urinary tract or nerves that control the urinary system (9.1%), psychological problems (8%), urinary tract infections (2.8%) and anemia, hereditary, pregnancy, and birth-related causes believed to be caused by small percentage of respondents. Schlomer et al. reported that parents in their study believed the most common cause of enuresis to be deep sleeper (56%), unknown (39%), laziness to wake up and go to the bathroom (26%) and small bladder size (21%) [19]. This perception did not change a lot from older studies that have mentioned that parents believed heavy sleeping, emotional problems, and small bladder size to be important causes of NE [19,20].

Participants also reported different practices in terms of modalities of treatment provided where behavioral modification was the most commonly used modality by 31.6%, followed by pharmacological treatment (29.6%), bedwetting alarm (6.8%), exercises to strengthen the bladder muscles (6.2%) and surgery reported by 1.5% only. We found that improvement of nocturnal enuresis on different types of treatment occurred in 43.6% of cases studied. In contrast to our results, Sherah et al. reported using medical treatment in 76% of case and Al-Zahrani et al. reported the treatment methods used to be: enuresis alarm, water restriction, medication, and awaking for voiding in 56.9%, 14.7%, 5.7% and 5.7% of cases, respectively [12,20]. Schlomer et al. reported that parents used some behavioral modifications like voiding prior to sleep (77%), limiting fluid intake at night (71%), and using bedwetting alarm (6%) [19].

A cross-sectional survey, performed in Primary Health Care Centers, found that out of 65 families that have children with NE, 38.7% was the frequency of bedwetting every night, 22.6% of the children were stressed as a result of new childbirth, 14% of the families did not feel a family load of having children with NE, 29% of the families did not try to treat their children because of their improvement with time, and 12% of the families that tried to treat their children used fluid restriction and waked their children up frequently at night [21].

Regarding risk factors of nocturnal enuresis among the studied children, our study found significant correlations between nocturnal enuresis and child age as the prevalence was significantly lower as children grew older (peak is 63.6% in 5-7 years old), and no significant relation was found between enuresis and gender (p = 0.104), which was also reported by a study done in Taif that has shown a prevalence of 7.33% and 8.42% in boys and girls, respectively [20]. In contrast to Bakhtiar et al. who reported the prevalence of nocturnal enuresis in boys (10.7%) to be higher than in girls (5.4%) (P = 0.009) [22]. However, there was a significant correlation with a parent suffering from the same condition in their childhood (p = 0.001). Another study reported that the prevalence of enuresis among boys was 1.65-fold greater than that of girls, and it was more common among children with positive familial history [17]. Another study found that the prevalence of enuresis was found to correlate well with age (p = 0.0001), but not correlate with gender (p > 0.05) [1]. In addition, Bakhtiar et al. reported statistically significant relationships between nocturnal enuresis and history of nocturnal enuresis in siblings (P = 0.023) and deep sleep (P = 0.007) amongst other factors [22].

## Conclusions

Our study reported that 31.2% of children suffer from nocturnal enuresis, but there were no significant correlations between nocturnal enuresis and child gender. There was a significant correlation with child age and family history of NE in parents or siblings. Behavioral modification therapy was the most provided treatment followed by pharmacological treatment and the improvement occurred in less than half of the cases. So, we recommend health education about the causes and risk factors in addition to encouraging prompt treatment and close follow-up to prevent associated self-shame and family stress. Further studies are needed to look in-depth into details of the modalities of treatment and how they are conducted and followed in addition to their effectiveness in Saudi children. Families' compliance with those modalities in Saudi culture is worth further investigation.
